# Current Eligibility Requirements for CGM Coverage Are Harmful, Costly, and Unjustified

**DOI:** 10.1089/dia.2019.0303

**Published:** 2020-02-21

**Authors:** John E. Anderson, James R. Gavin, Davida F. Kruger

**Affiliations:** ^1^ The Frist Clinic, Nashville, Tennessee.; ^2^ Emory University School of Medicine, Atlanta, Georgia.; ^3^ Division of Endocrinology, Diabetes and Bone and Mineral, Henry Ford He System, Detroit, Michigan.

**Keywords:** CGM, SMBG, Medicare, Eligibility, Medicaid, Insurance

## Introduction

Despite ongoing advances in medications, insulin delivery systems, and glucose monitoring technologies, diabetes control remains suboptimal in many individuals with this disease.^1^ In addition to the clinical consequences resulting from poor control is the ever-increasing financial burden on individuals, health systems, and society.^2^

In its latest report, the American Diabetes Association (ADA) estimated the total estimated cost of diagnosed diabetes in 2017 to be $327 billion.^2^ Although the direct cost of treating complications (hospitalizations, emergency room visits, and nondiabetes prescription medications) and the indirect costs associated with lost/reduced productivity account for ∼73.1% of the total diabetes cost, many public and private payers continue to focus much of their cost-cutting efforts on reducing the costs of diabetes supplies, which account for only 1.1% of the total cost.

Examples of these questionable efforts can be found in the restrictive eligibility criteria for coverage of continuous glucose monitoring (CGM). Specifically, the requirements are that eligible individuals must have type 1 diabetes (T1D) and be able to document routine performance of at least four fingerstick blood glucose tests per day.

The Centers for Medicare and Medicaid Services (CMS) explicitly requires this level of testing for Medicare beneficiaries to qualify for CGM coverage, as do 11 of 36 state Medicaid programs that provide CGM coverage. Requiring documentation of >4 blood glucose tests per day is clearly enigmatic given that the Medicare coverage policy only pays for three test strips per day for insulin-treated beneficiaries. Although Medicare coverage includes both T1D and intensively treated type 2 diabetes (T2D), many state Medicaid programs do not match such coverage. A comprehensive Internet search found that 13 state programs limit CGM use to individuals with T1D and documented history of 4 × /day blood glucose testing (Table 1). Three programs cover both T1D and insulin-requiring T2D but with the minimum blood glucose monitoring restriction. Four programs cover T1D with no blood glucose monitoring restriction, and only one state covers both type 1 and insulin-requiring diabetes with no blood glucose restriction. Eligibility for CGM in the remaining 14 State Medicaid programs is not specified or accessible according to our Internet search.

**Table 1.** Medicaid Coverage for Continuous Glucose Monitoring Use

Requirement	State medicaid programs
Type 1 with 4 × /day minimum SMBG requirement	Utah, Florida, Georgia, South Carolina, Michigan, New York, South Dakota, Mississippi, Maryland, Connecticut, Wyoming, Georgia, Florida
Type 1 with no SMBG requirement	Washington, Maine, Indiana, California, Oregon
Type 1 and insulin-requiring type 2 with 4 × /day minimum SMBG requirement	West Virginia, Iowa, Vermont
Type 1 and insulin-requiring type 2 with no SMBG requirement	Illinois
Unknown/not specified	Ohio, Idaho, Alaska, Montana, Colorado, Minnesota, Nevada, Kentucky, North Carolina, New Hampshire, New Mexico, Nebraska, Wisconsin, Tennessee

SMBG, self-monitoring of blood glucose.

These restrictions are also listed in the CGM eligibility criteria for two of the top five private insurers, Anthem and AETNA; however, United Health has somewhat liberalized the approach regarding self-monitoring of blood glucose (SMBG) frequency, stating that individuals must “have demonstrated adherence to a physician ordered diabetic treatment plan.” Descriptions of eligibility criteria for Humana, CIGNA, and Kaiser Permanente are not readily available to the consumer on the company websites. Moreover, United Health, Anthem, nor AETNA offer CGM coverage for individuals with T2D.

It is difficult to speculate about the reasons behind the lack of transparency in informing consumers affected by diabetes about their eligibility for CGM use. However, it is certain that valuable clinician time and staff resources are wasted when clinicians take the time to prescribe CGM only to find that their patients are ineligible.

As a result, many individuals who could improve their control with CGM use are denied coverage and, thus, limited to treatment regimens that fail to address their clinical and/or lifestyle needs. As such, uptake of CGM use has been slow. Data from the T1D exchange registry indicate that as of 2013–2014, only 9% of registry participants were using CGMs.^3^ In 2017 survey of 533 adults and 114 parents of children with T1D, 39.5% of respondents reported “not covered by insurance” as their primary reason for not trying CGM.^4^ A similar survey identified “cost of supplies” as the top reason for discontinuing CGM use.^5^ Therefore, even when CGM is covered, the out-of-pocket expense may be too high for many patients.

Although it is understandable and prudent for payers to limit use of technologies such as CGM to those populations most likely to benefit from its use, there is no evidence that frequent SMBG or type of diabetes is predictive of successful outcomes with CGM use. In short, these restrictions appear to be opinion based, not evidence based, as discussed in the following section. This is particularly disturbing in light of the plethora of evidence refuting these policies.

## Strong Evidence Supports the Benefits of CGM in All Individuals Treated with Intensive Insulin Regimens

Numerous large randomized trials have demonstrated the efficacy of CGM use in individuals with T1D^6–12^ and insulin-treated T2D.^13,14^ Specific benefits include reductions in A1c and glycemic variability; increased time in target glycemic range; decreased time in hypoglycemic range; and fewer hypoglycemic events. Importantly, CGM use has been shown to be particularly effective among those with frequent severe hypoglycemia and/or hypoglycemia unawareness.^10,11^

Recognizing the importance of glucose monitoring, especially for insulin-treated patients, the ADA recommends that most patients using intensive insulin regimens (multiple daily insulin injection [MDI] or insulin pump therapy) should assess glucose levels using SMBG or a CGM before meals and snacks, at bedtime, occasionally postprandially, before exercise, and when they suspect low blood glucose.^15^ Note that this evidence-based recommendation does not distinguish between T1D and T2D; rather, it focuses on the individual need of each patient based upon their treatment regimen. Nor, does it even mention the need for individuals to demonstrate a history of frequent fingerstick testing to achieve desired outcomes with CGM use. The reason why these restrictions are not stated in the recommendation is because they are not supported by any clinical evidence. As shown in the two largest T2D studies, REPLACE^13^ and DIAMOND T2D,^14^ there were no associations between baseline SMBG frequency among CGM users and outcomes.

The REPLACE study was a multicenter, open label, randomized controlled trial (RCT) that evaluated the impact of CGM use (FreeStyle Libre; Abbott Diabetes Care, Alameda, CA) compared with SMBG on hemoglobin A1c (HbA1c) and hypoglycemia among 224 adults with T2D who were treated with MDI or insulin pump.^13^ Although no significant between-group differences were seen in HbA1c change, CGM users spent significantly less time <70 mg/dL (*P* = 0.0006) and <55 mg/dL (*P* = 0.0014) compared with SMBG use. Importantly, a subgroup analysis of CGM users found no significant differences in these outcomes based on baseline SMBG frequency (Table 2).

**Table 2.** Change in Glycemic and Patient-Reported Outcomes Among Continuous Glucose Monitoring Users by Baseline Self-Monitoring of Blood Glucose Frequency in the REPLACE Study: ≥4 Versus <4 Tests/Day

	SMBG change from baseline		Adjusted mean change from baseline			
	SMBG frequency/day		SMBG frequency/day			
	≥4 (*n* = 90)	<4 (*n* = 59)	≥4 (*n* = 90)	<4 (*n* = 59)	Difference in adjusted means	*P*
HbA1c (%)	−0.21	−0.37	−0.29	−0.24	−0.05	0.6891
%Time <70 mg/dL (%)	−3.44	−2.23	−3.01	−2.90	−0.11	0.8497
%Time <55 mg/dL (%)	−1.77	−1.53	−1.63	−1.73	0.10	0.7012
Number of hypos <70 mg/dL	−0.32	−0.19	−0.27	−0.26	−0.01	0.9050
Number of hypos <55 mg/dL	−0.20	−0.18	−0.18	−0.22	0.04	0.3222
Treatment satisfaction (DTSQc^16^)	13.54	13.65	13.42	13.48	−0.06	0.9444

HbA1c, hemoglobin A1c.

In the DIAMOND T2D study, a randomized controlled clinical trial, investigators assessed the effects of CGM (Dexcom G4; Dexcom, San Diego, CA) use compared with SMBG on HbA1c and other measures among a cohort of 158 adults with T2D who were treated with MDI therapy.^14^ At baseline, the mean self-reported number of blood glucose tests for the CGM and SMBG groups was 3.3 and 3.2, respectively. At 6 months, the mean change in HbA1c was significantly greater in the CGM group (−1.0) compared with SMBG users (−0.6%), *P* = 0.005.

A subsequent analysis of the older T1D and T2D patients who participated in the DIAMOND trials showed a significant HbA1c reduction among CGM users versus control (−0.9% vs. −0.5%, *P* < 0.001).^17^ As with the REPLACE trial, subgroup analysis showed no apparent association between glycemic outcomes and baseline SMBG frequency. Interestingly, 33 (52%) of CGM users in this subgroup analysis reported SMBG frequency of <4 times per day at baseline. According to Medicare, these individuals (mean age 67 ± 5 years) were not eligible for CGM coverage.

## Use of Outdated and Inappropriate Evidence Is the “Fatal Flaw” in Policy Decision-Making

As demonstrated in the REPLACE^13^ and DIAMOND T2D^14^ studies, use of CGM in individuals with T2D who are treated with intensive insulin management confers significant clinical benefits. Moreover, our subgroup analyses showed no association between baseline SMBG frequency and outcomes.

Why, then, do so many payers continue to restrict CGM use only to individuals with T1D who test ≥4 times daily? Moreover, why is there such diversity among payers in their coverage policies? The answer may lie in the evidence used in their decisions and where they are getting it.

Payers often hire for-profit health research and technology organizations for guidance in coverage policy decisions. Their guidance is based on evidence gleaned from the literature. However, these organizations use varying methods for assessing the evidence used in their recommendations. For example, some may use the traditional model of five evidence levels, where systematic reviews and meta-analyses (SRMAs) are deemed the highest level and RCTs are considered the second highest. Conversely, the ADA considers SRMAs and RCTs to carry equal weight in grading the evidence used in their clinical guidelines.^15^ It is our position that the ADA model should be adopted by all policy decision makers when developing guidance recommendations and coverage policies that impact individuals with diabetes.

Although use of SRMAs provides insights into the comparative effectiveness of diabetes medications, it is also our opinion that they are inappropriate for assessing the value and utility of CGM, which are rapidly evolving. It is important to consider that conducting the outcome studies assessed in SRMAs and then publishing findings in a peer-reviewed journal is a lengthy process. By the time published reports from these clinical trials are published, analyzed, and compiled, those devices are irrelevant and outdated. Among the 12 available SRMAs that evaluated the clinical value of CGM in diabetes,^18–29^ only 2 reports were published after 2015,^18,19^ and only 1 evaluated studies published after 2015.^18^ In short, all of the available SRMAs assessed the efficacy of previous-generation CGM devices. These devices have since been replaced by more advanced systems that provide greater accuracy, longer sensor wear time, and factory calibration, which eliminates the need for daily calibration with fingerstick testing.

Importantly, we found no reference in any of the SRMAs suggesting that frequent SMBG should be a requirement for CGM eligibility. Although SMBG frequency at baseline was an inclusion criteria in most of the recent RCTs, eligibility requirements varied: ≥2 daily in DIAMOND T2D,^14^ ≥10 times weekly in REPLACE study,^13^ and ≥3 daily in IMPACT.^6^ It is our understanding that these criteria were included to support daily CGM calibration^30^ or to ensure a robust comparison between the CGM and SMBG study groups.^6,13^ No requirement for SMBG frequency was included in the DIAMOND T1D study.^7^

## Addressing the Problem

No one can dispute the need to effectively and efficiently utilize our tools and resources to improve diabetes outcomes. Unfortunately, cost-efficiency seems to trump clinical efficacy in coverage policies regarding CGM use. Although payers are ultimately responsible for their decisions, the companies that provide guidance to payers share much of the responsibility. It is unclear whether their recommendations for CGM eligibility are unwittingly driven by inappropriate methodologies used for evidence assessment or stem from the need (real or perceived) to provide their clients with guidance that, in the short term, reduces costs but does nothing to address the much greater cost of poorly controlled diabetes.

Whatever the reason, it is clear that different approaches are needed to more definitively assess the effects of behavior-based interventions such as CGM use. One approach would be to compare the percentage of study subjects who achieved significant clinical benefit, as was done in the recent analyses.^7,12,14,31^ This approach provides both an opportunity to identify and elucidate the characteristics of those patients who are most (or least) suited for the CGM use and the ability to more readily assess the economic benefits of the CGM in a more meaningful way. For example, it would be useful to know that 59% more patients can achieve clinically significant improvements in glycemic control (e.g., <7.5% HbA1c) using CGM versus SMBG, as shown in the DIAMOND T2D study.^14^ The potential cost savings can then be calculated within specific patient populations and compared with the incremental costs (if any) associated with the CGM use.

It is also important to consider the potential savings associated with reduced incidence and severity of the acute complications, particularly severe hypoglycemia. As reported in the recent HypoDE study, investigators found that the number of hypoglycemic events can be markedly reduced in individuals with impaired hypoglycemia awareness through use of real-time continuous glucose monitoring (rtCGM) compared with blood glucose monitoring.^11^ Investigators also found that rtCGM use resulted in a significant decrease in the frequency of clinical severe hypoglycemia and reduced glycemic variability, a known risk factor for hypoglycemia.^32,33^ Moreover, the most recent data from the T1D Exchange registry showed clear associations between increased adoption of CGM and decreased numbers of both hypoglycemia and diabetic ketoacidosis.^34^

Effective strategies to prevent moderate/severe hypoglycemia can lead to substantial short-term cost savings for patients and payers. For example, over a 5-year period, emergency department visits for severe hypoglycemia cost the U.S. health care system an estimated $600 million ($120 million per year),^35^ with an average cost of $1387 per visit.^36^ Denying individuals who would benefit from CGM use is not only clinically irresponsible but also it is clearly “penny wise and pound foolish.”

There is growing and compelling evidence that CGM coverage should be offered to all patients who can benefit from this technology regardless of diabetes type and history of SMBG use. The current restrictions, which are based on outdated evidence and questionable assessments, are not supported in the literature. Moreover, they ignore the burden frequent SMBG places on individuals. Policy makers who do not, themselves, have diabetes do not understand how the pain and frustration of frequent testing inhibit SMBG use among individuals who would like to monitor more frequently,^37^ a situation that could easily be overcome with CGM use. Given the growing prevalence of diabetes, the persistent preponderance of individuals with suboptimal glycemic control, and the exorbitant and largely preventable cost of diabetes complications, opinion-based constraints should not continue to supplant evidence-based clinical management.
